# The Role of Biofunctional Polymers in Polymer–Drug Conjugates: From Passive Carriers to Therapeutically Active Platforms

**DOI:** 10.3390/pharmaceutics18040419

**Published:** 2026-03-29

**Authors:** Camilla Passi, Armin Walter Novak, Marc Schneider, Sangeun Lee

**Affiliations:** 1 Department of Pharmacy, Pharmaceutical Materials and Processing, Saarland University, Campus C4.1, 66123 Saarbruecken, Germany; camilla.passi@uni-saarland.de; 2 Department of Pharmacy, Biopharmaceutics and Pharmaceutical Technology, Saarland University, Campus C4.1, 66123 Saarbruecken, Germany; armin.novak@uni-saarland.de; 3 PharmaScienceHub (PSH), 66123 Saarbruecken, Germany; 4 Helmholtz Institute of Pharmaceutical Research Saarland (HIPS), Helmholtz Centre for Infection Research (HZI), Campus E8.1, 66123 Saarbruecken, Germany

**Keywords:** polymer–drug conjugates, bioactive polymers, stimuli-responsive systems, drug delivery, poly-lysine, gelatin, hyaluronic acid, chitosan, biodynamers

## Abstract

Polymer–drug conjugates (PDCs) represent an advanced drug delivery strategy designed to address critical limitations of conventional therapeutics, including poor water solubility, rapid systemic clearance, and off-target toxicity. By covalently linking therapeutic agents to polymeric carriers through rationally designed linkers, PDCs enable improved pharmacokinetic profiles, enhanced stability, and controlled drug release. This review provides a comprehensive overview of the key design principles governing PDC systems, with a particular focus on the role of biofunctional polymers. Essential parameters for polymer selection, including biocompatibility, biodegradability, molecular weight, and functional group availability, are discussed in relation to their influence on drug loading, release kinetics, and biological performance. In addition, both natural and synthetic polymers are evaluated for their ability to improve solubility, modulate biodistribution, and reduce systemic toxicity. An overview of stimuli-responsive PDCs is provided, including pH-, redox-, and temperature-sensitive systems, which enable site-specific and spatiotemporally controlled drug release in response to pathological microenvironments. We emphasize the special role of bioactive polymers such as poly-lysine, hyaluronic acid, chitosan, and gelatin for their intrinsic biological activity, including receptor-mediated targeting, antimicrobial activity, and synergistic therapeutic effects. These properties support the development of dual-active conjugates with enhanced specificity and efficacy. Overall, this review underscores the transition of polymers from passive carriers to active therapeutic components and outlines current challenges and future perspectives for the clinical translation of next-generation PDCs.

## 1. Introduction

Despite major advances in pharmaceutical development, the clinical translation of many therapeutic agents remains hindered by critical limitations in drug delivery. Poor water solubility, rapid systemic clearance, low bioavailability, and non-specific biodistribution often compromise therapeutic efficacy and increase systemic toxicity [1,2,3]. These challenges are particularly pronounced in the treatment of chronic diseases such as cancer, autoimmune disorders, and infections, where precise delivery and controlled release of therapeutics are essential [4,5,6,7]. To address these limitations, polymer–drug conjugates (PDCs) have emerged as a promising class of delivery systems that can enhance the pharmacokinetics, stability, and target specificity of therapeutic agents [8,9]. By covalently attaching payloads, such as therapeutic or targeting agents to polymeric carriers, PDCs allow for prolonged circulation, improved solubility, and controlled drug release, while also enabling passive or active targeting of specific tissues [10,11,12].

**Figure 1:**
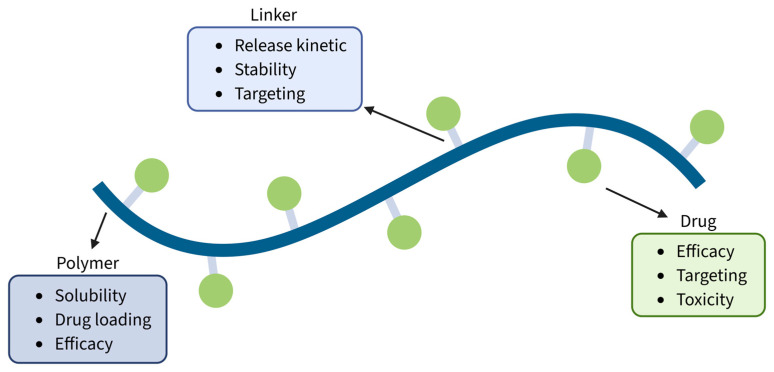
Schematic illustration of a polymer–drug conjugate consisting of a polymeric backbone, a linker controlling essential physicochemical properties, and a conjugated drug.

Structurally, PDCs consist of three key components: a therapeutic agent, a polymer backbone, and a linker that connects them (Figure 1) [13]. The therapeutic agent is typically a small drug whose activity is retained or activated upon release from the conjugate. Small hydrophobic anticancer drugs like paclitaxel, doxorubicin, gemcitabine, and cisplatin are often conjugated to polymeric carriers to enhance tumor accumulation, improve intracellular uptake, and reduce systemic toxicity [14,15,16,17,18]. The choice of drug is, therefore, a critical design parameter, as its physicochemical and biological properties directly shape the overall behavior of the PDC. Parameters such as hydrophobicity, molecular weight, and functional groups available for conjugation determine not only the feasibility of the coupling reaction but also the self-assembly behavior of the final conjugate, influencing nanoparticle size, loading efficiency, stability, and release kinetics [19]. Drugs with appropriate reactive moieties (e.g., amines, hydroxyls, and carboxylates) enable stable yet cleavable linkages, while their inherent potency dictates the required loading levels for therapeutic efficacy [20]. The drug’s pharmacodynamics and intracellular trafficking can also affect targeting ability: for example, drugs prone to rapid efflux may benefit from conjugation strategies that exploit receptor-mediated endocytosis to enhance retention [21].

These drug-dependent considerations also influence the selection of the linker, which must be chemically compatible with both components and capable of providing controlled release. The linker can be designed to be cleavable under specific physiological conditions—such as acidic pH, enzymatic activity, or redox environment—ensuring site-specific or stimuli-responsive drug release [22,23]. Arroyo-Crespo et al. developed a family of PDCs based on the conjugation of poly-L-glutamic acid (PGA) with doxorubicin (DOX) and aminoglutethimide (AGM), using different amino acid-based linkers (Gly, Phe, Leu) [24]. By changing the length of the linker and the type of amino acid, the performance and efficacy of the resulting PDCs changed accordingly, due to the differences in spatial conformation and flexibility of the conjugate, showing the impact of the linker on the properties of the final delivery system [25]. Similarly, Xu et al. investigated the effect of the linker in the formation and final activity of a xyloglucan–doxorubicin–mitomycin C conjugate [26]. By introducing different linkers (amide or peptide bond), they could synthesize PDCs with adjustable drug release rate and cytotoxicity as a result of the different cleavability of the linkers. Alongside these reports, the importance of linker selection and its influence on the properties of macromolecular delivery systems has been highlighted [27,28,29].

Finally, the choice of polymer plays a critical role in the development of PDCs, as it directly influences the conjugate’s pharmacokinetics, biocompatibility, degradation behavior, and targeting ability. This review provides an overview of the three main categories of polymers used in the design and synthesis of PDCs. Initially, polymers are selected according to their physicochemical and biological properties, as these characteristics strongly influence the safety, stability, and therapeutic performance of the conjugate. Polymers exhibiting minimal toxicity and high tolerance within biological systems, commonly referred to as biocompatible polymers, are typically preferred for drug conjugation. Their favorable safety profile makes them particularly suitable as carriers for the systemic delivery of therapeutic agents.

Within this class, certain polymers are not just simple carriers, but they play a functional role in the final system. Some of these biofunctional polymers are capable of responding to stimuli present in the surrounding environment, such as variations in pH, temperature, enzymatic activity, or reactive oxygen species (ROS) levels, enabling controlled and site-specific drug release.

In addition to stimuli-responsive systems, another important category within the biofunctional polymers is represented by the bioactive polymers, which possess intrinsic biological activity. Biologically active polymers, such as hyaluronic acid, chitosan, gelatin, and poly-lysine, have gained significant interest due to their intrinsic bioactivity and receptor-specific interactions, which can further enhance therapeutic outcomes. In this context, the polymer component plays a dual role: it serves as a structural scaffold that governs the physicochemical properties of the conjugate and additionally contributes to the therapeutic effect through biological interactions. However, some of the polymers described in this work inherit a combination of properties described in the three main sections of this work and are therefore discussed in several paragraphs, each highlighting the specific property and its contribution to either biocompatibility, stimuli-responsiveness, or intrinsic biological activity.

**Figure 2:**
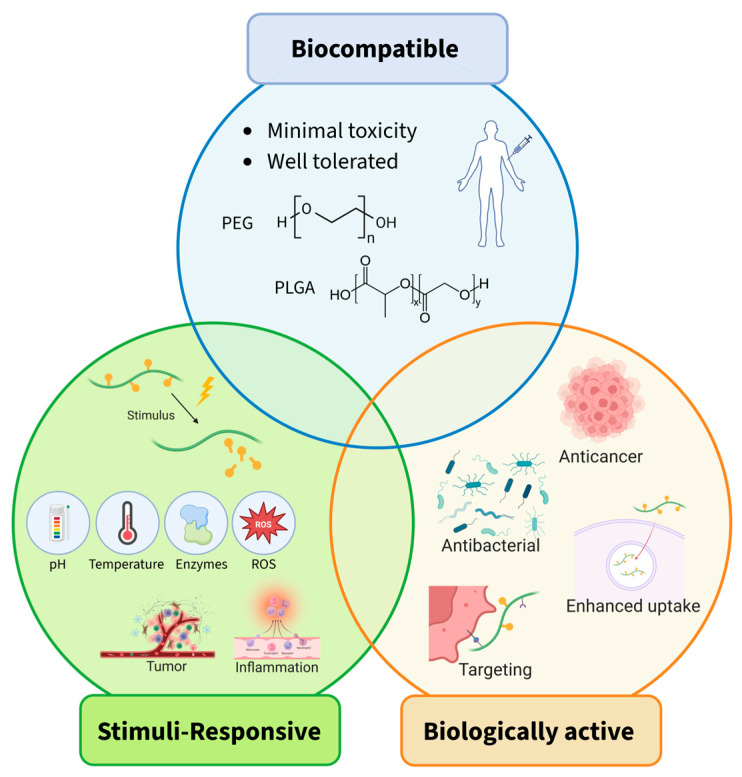
Schematic illustration of the three main categories of polymers used in PDC design. Biocompatible polymers, such as PEG and PLGA, show low toxicity and are highly tolerated by the organism. Stimuli-responsive polymers can react to external triggers, i.e., pH, temperature, enzymes, and ROS, to selectively deliver a drug to its target. Finally, biologically active polymers have intrinsic activity or the ability to improve uptake and targeting, actively contributing to the final performance of the PDC. The three categories can overlap, as biocompatible polymers can show stimuli-responsive properties or intrinsic activity by their own. At the same time, biologically active polymers can also provide a stimulus-responsive delivery of drugs, acting both as a responsive and active carrier.

This review explores the emerging trends in PDCs, with a particular focus on the pivotal role of biologically active polymers in designing novel therapeutic platforms. To put bioactive polymers into perspective, this work will introduce important factors in the selection of polymers for conjugation, as well as highlight various polymers used in PDCs with a focus on their interactions and activity within biological environments (Figure 2). Subsequently, the aforementioned biologically active polymers will be illustrated by showcasing conjugation methods, linkable drugs, and the intrinsic efficacy of the polymers.

## 2. Polymer Selection in Polymer–Drug Conjugates

In the design of PDCs, the choice of polymer fundamentally governs the physicochemical and biological attributes of the resulting conjugate. Several selection criteria must be considered to align polymer properties with the desired performance of the PDC. The factors influencing the final properties of PDCs are summarized in Table 1 and more extensively discussed in this chapter.

### 2.1. Biocompatibility

Among the factors to consider in polymer selection in PDC, biocompatibility and biodegradability stand out as essential requirements: biocompatibility ensures that the polymer interacts safely with biological systems, minimizing immunogenicity, cytotoxicity, and adverse tissue responses, while biodegradability enables the polymer to degrade into non-toxic byproducts that can be cleared by endogenous pathways, thereby avoiding secondary toxicity once the cargo has been released [42,43,44]. In addition to its own biocompatibility, the polymer is often used to lower the toxicity of small drugs, improving their biocompatibility.

Especially in cancer treatment, small antitumor agents, including doxorubicin (DOX), camptothecin (CPT), and paclitaxel (PTX), are conjugated to biocompatible polymers to successfully lower undesired toxicity and enhance the efficacy once the target is reached [47,48]. Zhou et al. conjugated DOX, a potent anticancer drug that has numerous adverse effects, to poly(2-ethyl-2-oxazoline) (PEtOZ) to enhance the efficacy of the drug and reduce its toxicity, thus increasing the biocompatibility of the system. PEtOZ showed good hydrophilicity and excellent biocompatibility. The resulting conjugate, DOX-PEtOZ, was tested for cytotoxicity on healthy mammalian cells as well as on cancer cells. DOX-PEtOZ showed up to an 8-fold decrease in the IC_50_ on healthy cells compared to free DOX, while retaining the ability to inhibit the growth of cancer cells. Additionally, the conjugate showed higher selectivity towards cancer cells, with a selectivity index up to 2.7 times higher than that of free DOX [49]. This can be explained by the change in hydrophilicity and charge of DOX after conjugation, resulting in a different interaction of DOX with mammalian cell membranes. Overall, these findings underscore how the use of a biocompatible and biodegradable polymer carrier in a PDC cannot only reduce systemic toxicity of the free drug but also enhance its therapeutic selectivity and efficacy, thereby reinforcing the strategic value of polymer choice in improving the safety and performance of drug-delivery systems.

### 2.2. Solubility

In addition to biocompatibility and biodegradability, the choice of polymer must also account for its ability to enhance the aqueous solubility of the conjugated drug, since many small-molecule therapeutics suffer from poor water solubility that limits formulation and bioavailability [50]. By selecting a hydrophilic polymer carrier, the conjugation process can markedly improve the dissolution behavior of a hydrophobic drug, thereby widening its clinical applicability and enabling more reliable systemic delivery [25,30].

An example of how drug solubility can be improved by conjugation with a polymeric carrier is reported by Nagahama et al. [51]. In the study, curcumin is used as an anticancer agent, but its solubility represents a limitation for its clinical applications. Dextran was, therefore, used as a vehicle to increase curcumin’s solubility by conjugation. The resulting conjugates showed approximately 2.4 × 10^3^–1.9 × 10^4^-fold higher solubility in water compared to the free drug. Additionally, the curcumin–dextran conjugates showed the ability to self-assemble into nanoparticles (NPs), obtaining selective cellular uptake in cancer cells via endocytosis, due to a selective binding between curcumin molecules in the nanoparticles and cancer cell-specific membrane proteins [51]. Similarly, one of the most common anticancer drugs, PTX, available on the market as Taxol^®^, was conjugated to poly-L-glutamic acid (PGA) to overcome the limitations connected to the low solubility of this drug. Because of its poor solubility in water, Taxol^®^ has to be administered via an infusion for up to 24 h, containing a mixture of polyethoxylated castor oil (Cremophor^®^) and ethanol as additives [52]. To overcome the solubility issues of Taxol^®^ and the hypersensitivity reaction associated with its formulation, PTX was conjugated to PGA to form a PDC, which reached clinical trials under the name of Xyotax (later named Opaxio). Xyotax improved the water solubility of PTX and allowed for shorter infusion times (10–20 min) with enhanced efficacy compared to Taxol^®^ [53,54]. The notable increase in aqueous solubility achieved by forming a PDC through conjugating a hydrophilic polymer to a poorly soluble drug not only facilitates formulation and systemic delivery of the drug but also highlights solubility as a critical parameter in polymer selection for PDCs.

### 2.3. Pharmacokinetics

In addition to addressing the challenge of poor solubility in aqueous environments, conjugation of small-molecule drugs to an appropriate polymer also enables the modulation of pharmacokinetic and pharmacodynamic behavior of the resulting system. For example, PDCs have been shown to extend systemic circulation times, alter biodistribution and cellular uptake pathways, reduce off-target exposure, and thereby refine both the therapeutic efficacy and safety profile of the parent drug [45,46].

This can be further illustrated by the previously mentioned example of Xyotax. By formulating this PTX conjugate, not only was the low water solubility of the drug enhanced, but it also resulted in improved pharmacokinetics, including longer circulation time and enhanced tumor accumulation [55]. Among the numerous polymers used for PDC formulation, poly(ethylene glycol) (PEG) is often used to modify the physicochemical properties and pharmacokinetics of a drug. An interesting approach to how PEGylation is used to modify the pharmacodynamics of the drug is given by Dodd et al., who conjugated PEG to haloperidol [56]. The objective of the study was to investigate the ability of PEG to restrict drugs to the maternal bloodstream and limit the fetal transfer. The PEG–haloperidol conjugate was prepared using PEG at two different molecular weights (MWs): 6 and 2 kDa. The conjugates showed a 4.2 and 45-fold decrease in the cellular uptake by placenta explants after 24 h, respectively, demonstrating the use of PEG as a drug carrier and as a promising way to reduce or prevent the transfer of drugs across the placenta [56].

### 2.4. Molecular Weight

Following up on this latest example, molecular weight occurs as another important factor in the synthesis and development of PDCs. Polymers with a higher molecular weight (>50 kDa) tend to show longer plasma circulation times as well as increased tumor accumulation [31,32,33].

In a study performed by Etrych et al., various star polymer conjugates were synthesized by grafting hydroxypropylmethacrylate precursor molecules onto poly(amidoamine) dendrimers and conjugating them with DOX [57]. In a comparison of these PDCs, ranging from 200 to 1000 kDa, a superior treatment efficiency in tumor-bearing mice could be observed for the high-MW conjugates over the non-dendritic DOX conjugate with lower MW. Additionally, the higher the MW of the polymers, the more long-term survivors were discovered. The authors concluded that the sweet spot for passive tumor accumulation is located in a range between 200 and 600 kDa [57].

Despite PDCs with higher MW displaying enhanced tumor accumulation and decreased renal clearance, conjugates with lower molecular weight can still be of interest in the development of PDCs. It could be shown that low-MW hyaluronic acid (HA) conjugates of the anticancer drugs CPT and PTX possessed a superior in vitro anticancer activity compared to high-MW HA conjugates, likely due to a higher conjugation efficiency of the drugs for the smaller molecules of HA [58]. Overall, the molecular weight of the polymeric scaffold needs to be chosen carefully, as higher tumor targeting and longer plasma circulation times do not always concur with a higher treatment efficiency eventually. 

### 2.5. Charge

The charge of a polymer is an important factor in the development of PDCs, as it is able to alter the conjugate’s cellular uptake, cytotoxicity, and, in some cases, even adds intrinsic, charge-dependent efficacy to the final compound [34,35]. A PDC synthesized by Soukos et al., consisting of the photosensitizer, chlorin e6, and poly-L-lysine, possessing either cationic, anionic, or neutral charges, showed significant differences regarding their cellular uptake, intracellular localization, and efficacy [59]. For the cell uptake, the cationic conjugate displayed a 5.7–4.7-times higher uptake compared to the anionic conjugate, with both charged PDCs being superior to the neutral conjugate. Fluorescence microscopy revealed an internalization of anionic and neutral PDCs into the membrane and cell organelles, while the cationic PDC was localized in the cytoplasm. Contrary to these findings, the neutral PDC possessed a much higher phototoxic efficacy compared to its positively and negatively charged PDCs, drastically reducing the cell number after a 24 h incubation period [59]. Taken together, these results showcase that the polymer charge is an important, yet complex parameter of the therapeutic performance of PDCs, as increased cellular uptake alone does not guarantee improved photodynamic efficacy or reduced cytotoxicity. As a result, PDCs with different charges might, therefore, be carefully considered for specific targets or applications in photodynamic cancer therapy [60].

### 2.6. Polymer Architecture

An additional consideration in selecting a polymer for the formulation of a PDC is the polymer architecture, encompassing both the molecular composition and the overall chain topology [61]. Polymers may consist of a single homopolymer chain or be composed of block copolymers. They can further adopt linear or branched configurations, including brush or star-shaped polymers and dendrimers [36,62,63]. In PDC formation, polymer architecture controls not only its fate in the body (circulation time, excretion, etc.) but, more importantly, drug conjugation efficiency and release.

Block copolymers are widely employed in PDC design as they enable the integration of complementary properties from different polymer blocks, thereby allowing fine control over physicochemical characteristics such as solubility, drug conjugation, and release behavior, as well as facilitating the incorporation of stimuli-responsive functionalities [36,37,38,39]. Branched polymers show advantages as well, compared to the linear ones, including high density of functionalities, enhanced encapsulation ability, and higher solubility [64,65,66,67,68]. The impact of the polymer architecture on its in vivo behavior and efficacy is illustrated by the N-(2-hydroxypropyl) methacrylamide–doxorubicin conjugates (HPMA–DOX) examined by Etrych et al. [57]. By comparing linear and star-shaped HPMA carriers with similar molecular weight, they found that the more compact and rigid star-shaped polymer resulted in substantially prolonged blood retention and higher tumor accumulation. Additionally, while low-MW linear conjugates showed only modest tumor exposure and limited survival benefit, star-shaped carriers achieved significantly enhanced tumor deposition and, at higher MW, even complete tumor regression in tumor-bearing mice [57]. Thus, polymer architecture provides a powerful handle to modulate in vivo behavior and efficacy of PDCs.

### 2.7. Functional Groups

A key determinant in the design of PDCs is the selection of the polymer based on the functional groups present along the backbone. These moieties govern the chemical reactivity of the carrier and dictate how the polymer interacts with both the payload and the surrounding environment [69,70,71]. In the context of PDCs, the functional groups available for modification determine which drugs can be covalently attached, the efficiency and selectivity of the coupling reaction, the achievable drug-loading capacity, and the subsequent release kinetics [40,41]. Commonly employed polymers often present primary amines (-NH_2_), hydroxyl (-OH), or thiol (-SH) groups, which serve as versatile anchoring points for drug conjugation [72]. The chemical nature of these groups determines whether direct coupling with the drug is feasible or whether a linker is required to achieve stable attachment. Notably, the functional groups introduced or transformed during conjugation can significantly influence the overall physicochemical properties of the carrier (e.g., net charge, hydrophilicity) or its ability to respond to environmental stimuli, thereby further modulating the pharmacokinetics and therapeutic behavior of the final PDC [40].

**Figure 3:**
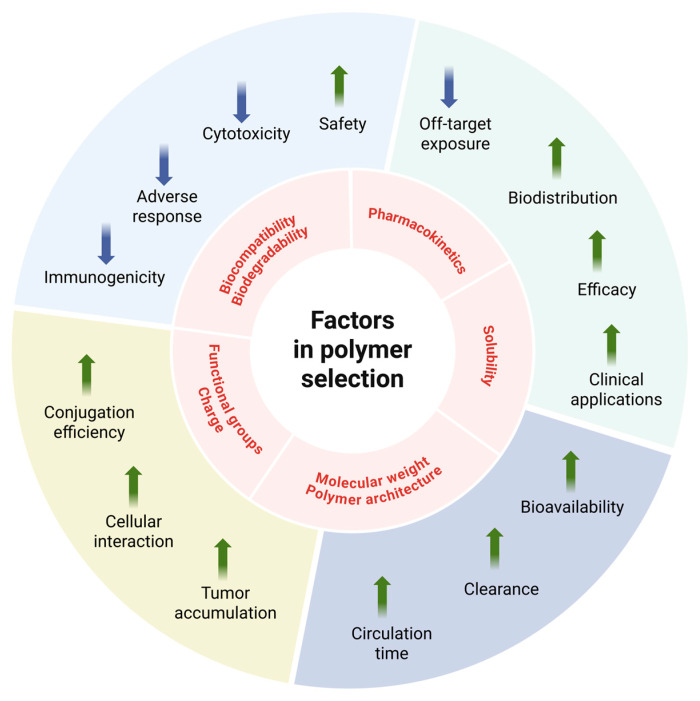
Overview of important factors in the selection of polymer candidates, displaying their various influences on the resulting PDC. Core polymer properties, including biocompatibility and biodegradability, pharmacokinetics, solubility, molecular weight and polymer architecture, and functional groups/charge, govern downstream therapeutic outcomes. These properties can act in a complementary manner and influence one another, collectively shaping the overall therapeutic outcome of the PDCs. Biocompatibility and biodegradability reduce immunogenicity, adverse responses, and cytotoxicity, thereby improving the overall safety of the system. Pharmacokinetics and solubility influence biodistribution, minimize off-target exposure, and enhance efficacy and clinical applicability of the resulting PDCs. Molecular weight and architecture determine circulation time, clearance, and ultimately bioavailability. Functional groups and polymer charge modulate conjugation efficiency, cellular interactions, and tumor accumulation, enabling more effective and selective drug delivery. Collectively, these interconnected parameters dictate the therapeutic performance and translational potential of PDC systems.

In summary, the factors illustrated in Figure 3 showed a significant influence on the final efficacy and potency of the synthesized PDCs and thus should be thoroughly investigated during the development process. Nevertheless, it is important to mention that most of these factors are co-dependent on each other and therefore a thoughtful consideration of the respective PDC is essential. 

## 3. Biocompatible Polymers

Biocompatibility is a fundamental requirement for polymers used in PDCs, meaning that the polymer and its degradation products are bioinert and do not trigger toxicity, inflammation, or immune responses [44,73,74]. As a result, the PDCs can safely circulate within the body, deliver the therapeutic agent to the target site, and degrade or be eliminated without adverse effects [8,75]. Beyond safety, biocompatible polymers in PDCs provide a stable and tunable platform for drug conjugation. They enable chemical modification, high drug conjugation, with favorable solubility and degradation profiles, thereby shaping pharmacokinetics and biodistribution to improve therapeutic efficacy and minimize off-target effects [8,76,77,78].

Among the biocompatible polymers, natural polymers have gained attention due to their inherent biodegradability, low toxicity, and structural resemblance to components of the extracellular matrix [78]. These attributes enable them to interact favorably with biological systems, making them ideal candidates for safe and efficient drug conjugation strategies. Alginate and dextran are representative examples widely used as biocompatible carriers. While natural polymers offer intrinsic biocompatibility, their structural variability and limited mechanical stability can sometimes restrict their use in precisely engineered drug delivery systems. Synthetic biocompatible polymers have been developed to provide greater control over composition, molecular weight, and functionalization, allowing fine-tuning of degradation rates and pharmacokinetic behavior. These materials retain the safety profile of natural polymers while offering enhanced reproducibility and design flexibility. This section highlights representative natural and synthetic polymers used for PDCs formation and discusses their roles in drug delivery.

### 3.1. Alginate

Alginate is an anionic copolymer naturally present in seaweed, composed of 1,4-linked β-D-mannuronic acid and α-L-glucuronic acid. As a biocompatible and biodegradable polymer, alginate is widely used as a drug carrier in drug delivery systems, not only for its low toxicity but also for its chemical versatility. Its numerous carboxyl groups are available for modification, providing opportunities for facile drug conjugation and crosslinking. These properties position alginate as an effective drug carrier, particularly for cancer therapy [79,80,81,82].

As an example of alginate’s potential in cancer therapy, the polymer was conjugated to cisplatin in order to increase the solubility of the drug, which represents a main limitation in the chemotherapeutic agent’s clinical use, allowing its incorporation into modified liposomes (CS-EGF-Lip) to target epidermal growth factor receptor (EGFR) expressing tumors. The CS-EGF-Lip showed a 3.7-fold inhibition of SKOV3 spheroids’ growth, and a 3.8-fold induction of apoptosis in SKOV3 compared to free cisplatin. Additionally, the formulations were tested in vivo using the xenograft model, where CS-EGF-Lip could effectively induce an antitumor response in SKOV3 cancer cells [83]. A similar targeting system was obtained by using folic acid as a folate receptor targeting agent [84]. After the conjugation of folic acid to alginate, the product was crosslinked using disulfide bonds to form a redox-responsive and tumor-targeting microgel for the loading and targeted delivery of DOX. Cell uptake studies proved selective uptake of the microgel into cancer cells MDA-MB-231, due to the specific interaction of the folic acid with the folate receptors of cancer cells, while flow cytometry analysis showed that DOX-loaded microgels can strongly induce cell apoptosis, indicating the potential of alginate-based microgels as anticancer drug carriers [84].

### 3.2. Dextran

As previously mentioned, alongside alginate, dextran is another important polymer commonly used in drug delivery. Dextran is a water-soluble, branched glucan composed primarily of α-1,6-linked glucose units with occasional α-1,3 branches [85]. Its abundant hydroxyl groups allow for straightforward chemical conjugation of drugs and targeting ligands [86,87]. Additionally, dextran can be engineered to degrade under specific physiological conditions, enabling controlled and site-specific release, which will be further discussed in the next paragraph [88,89]. Its intrinsic biocompatibility and capacity to reduce immunogenicity make it a valuable polymer for PDC formulations requiring increased drug solubility, extended drug retention, and reduced clearance [90,91,92,93].

In this context, mesalamine (5-aminosalicylic acid) (5-ASA) was conjugated to dextran for the treatment of inflammatory bowel disease [94]. 5-ASA is an anti-inflammatory drug suffering from unwanted absorption in the upper GI tract and rapid clearance. The conjugate, 5-ASA-dextran, was proven to bypass the acidic environment of the stomach, the duodenum, and jejunum without modification to subsequently reach the targeted area, the distal ileum and proximal colon, where specific enzymes allow the release of 5-ASA [94]. The study displayed how the conjugation of a small drug to a polymer like dextran has the potential to overcome the problems associated with rapid blood clearance and drug-related side effects [94,95].

A different example, making use of the numerous hydroxyl groups of dextran available for modification, is given by Varshosaz et al. by developing a targeting system via dextran modification with folic acid as a targeting agent and all-trans-retinoic acid (RA) as an antiproliferative agent [96]. Folic acid is used in drug delivery for its ability to target the FR-α and FR-β receptors, considered as cellular markers because of their overexpression in solid tumors and in leukemia [97]. RA is used in cancer treatment for its role in the pathway of regulation of cell differentiation and inhibition of cell proliferation [98]. Additionally, RA showed the ability to upregulate the FR-β receptor in acute myelogenous leukemia cells and, after conjugation to dextran, it played an important role in determining the properties of the final PDC [99]. The conjugation of dextran and RA enabled micelle formation of the resulting conjugate. Furthermore, the degree of RA conjugation influenced micelle formation, with higher conjugation levels increasing hydrophobicity and consequently decreasing the CMC value. The folate-targeted micelles loaded with DOX increased cellular uptake by ~20% and significantly enhanced antiproliferative activity against KG-1 leukemia cells in vitro, reducing the IC_50_ approximately 2-fold compared to free DOX. The final dextran–folic acid–RA conjugate represents a potential strategy for a targeting system that can improve the treatment outcome by increasing drug efficacy and reducing toxicity [96].

### 3.3. Poly(ethylene glycol)

Poly(ethylene glycol), PEG, is a synthetic, biocompatible polymer, the most extensively used in drug delivery, and frequently employed in PDCs. PEGylation involves activation of the terminal hydroxyl groups, making them available for conjugation with numerous drugs. This technique provides a straightforward approach to modify the pharmacokinetics and pharmacodynamics of the drug while simultaneously minimizing non-targeted uptake and extending circulation time through its stealth properties, reducing immune recognition [100,101,102,103,104]. These benefits can play an important role, particularly in cancer treatment, to promote tumor accumulation through passive targeting and minimize the side effects of the chemotherapeutic drugs.

In a different study, Veronese et al. synthesized a series of PEG-DOX conjugates using linear and branched PEG at different molecular weights to observe the influence of the polymer structure on the properties of the final conjugate. All PEG-DOX conjugates showed a more than 10-fold decrease in toxicity compared to free DOX, in addition to prolonged plasma clearance and enhanced tumor targeting. Furthermore, the highest molecular weight PEG showed the longest plasma retention time, resulting in better tumor targeting compared to the other molecular weights [68]. Similarly, PTX, a microtubule inhibitor, was modified with PEG to retain the drug in the lungs and, therefore, increase the efficacy of the anticancer treatment. The conjugation of PTX with PEG at two different molecular weights (6 kDa and 20 kDa) resulted in a 100-fold increase in the Maximum Tolerated Dose (MTD) tested on mice, compared to the free drug, thus decreasing the toxicity of the drug. Additionally, the 20 kDa conjugate showed a higher retention in the lungs, where up to 43% of the initial dose was found after 48 h post-intratracheal instillation, demonstrating the superior antitumor efficacy of the conjugates in Lewis lung carcinoma treatment [105]. As demonstrated in these studies, PEG not only enhances therapeutic efficacy when used as a PDC backbone but also serves as a valuable research platform. Its synthetic nature enables precise control over molecular weight and structure, facilitating systematic investigation of polymer backbone effects in PDC systems.

### 3.4. Poly(lactic-co-glycolic Acid)

Together with PEG, the synthetic copolymer poly(lactic-co-glycolic acid) (PLGA) is one of the most widely used polymers for drug delivery. Its broad field of applications includes cancer therapy, immunology, and anti-infective treatment [106,107,108]. Due to the composition of its two endogenous monomers, lactic acid (LA) and glycolic acid (GA), the copolymer possesses excellent biocompatibility as well as biodegradability in vivo [109]. Moreover, as a copolymer, the ratio between LA and GA has a crucial impact on the polymer’s properties, such as degradation behavior, hydrophilicity, and cellular uptake [110,111,112]. It is, therefore, important to carefully select the specific type of PLGA with a determined LA:GA ratio for the suitable application [113]. Furthermore, PLGA displays good accessibility for chemical modification, offering various opportunities for its use as a PDC [114].

In a study carried out by Wang et al., the broad-spectrum antibiotic tetracycline was linked to PLGA via an esterification between the polymer’s carboxyl groups and hydroxyl moieties of tetracycline [115]. Subsequently, the PDC was used to form nanoparticles, which were loaded with simvastatin for the treatment of osteoporosis. The nanoparticles composed of the PLGA–tetracycline conjugate were compared to pure PLGA nanoparticles and showed an enhanced bone-binding capacity as well as an increase in in vivo efficacy in rats over the blank PLGA particles.

Further, PLGA–drug conjugates are not limited to parenteral applications but can also be used for transdermal administration. For this experiment, the amino-monosaccharide glucosamine was covalently bound to PLGA using 1-ethyl-3-(3-dimethylaminopropyl)carbodiimide (EDC) as a crosslinker [116]. The hydrophobic PLGA backbone of the PDC enabled transdermal permeation of the hydrophilic drug glucosamine, forming self-assembling nanoparticles that imitate the lipid bilayer of the skin. This system could resemble an effective treatment option for inflammatory diseases, e.g., rheumatoid arthritis or osteoarthritis, in the future.

### 3.5. Polyoxazoline

Polyoxazolines (POZs) represent a versatile class of polymers, characterized by an oxazoline monomer structure to which different side chains and drugs can be conjugated [117]. In particular, growing evidence shows that repeated administration of PEGylated therapeutics can induce anti-PEG antibodies, resulting in reduced efficacy and increased adverse reactions. Together with reported concerns regarding stability and tissue accumulation, these limitations have collectively accelerated interest in POZ as an alternative polymer platform [118,119,120].

Among the class of POZ, 2-polyoxazolines represent the most commonly used ones, displaying good biocompatibility and sufficient solubility for pharmaceutical applications [121,122]. The solubility of the polymer can be altered by the introduction of alkyl side chains of varying lengths, with poly(2-methyl-2-oxazoline) (PMeOZ) and poly(2-ethyl-2-oxazoline) (PEtOZ) showing good water solubility at room temperature, while leading to a more hydrophobic polymer than the original compound. This can be used to adjust the hydrophilicity of the polymer to match that of conjugated drugs or to balance the molecule’s overall hydrophilic properties [121]. This ability, specifically, makes POZ an interesting polymer for the synthesis of PDCs, which we will highlight with a few selected samples.

Park et al. used POZ with alkyl side chains of different lengths (C_1_–C_4_) for the conjugation of the angiotensin-converting enzyme inhibitor benazepril (BNZ) [123]. After chemically modifying BNZ with thionyl chloride to obtain 2-mercaptoethyl benazepril, the drug was conjugated to POZ via thiol-ene photo-conjugation. Due to its lability, the resulting ester linkage enabled a sustained release of BNZ. Depending on the length of the used alkyl chain, the in vitro drug release could be controlled from days up to several months, with PMeOZ showing the fastest release and POZ using butyl functions displaying the most sustained [123].

Another example focuses on the synthesis of PEtOZ of different molecular weights, conjugated with the anticancer drug, DOX [124]. The polymers were functionalized with additional methyl ester groups, which were subject to hydrazinolysis, resulting in hydrazides that were used for the conjugation of DOX. The two produced polymers had MWs of 20 and 40 kDa, respectively, and enabled an acid-triggered drug release via the degradable hydrazone bonds. While the MW of the PDCs had no significant impact on the release kinetics, the 40 kDa conjugate offered prolonged blood circulation time, superior antitumor efficacy, and better tumor accumulation compared to its counterpart with a lower MW [124]. Through their accessible modifications in terms of hydrophobicity and molecular weight, together with their excellent biocompatibility, polyoxazolines exemplify an interesting class of polymers for PDC synthesis.

Overall, a wide variety of biocompatible polymers have demonstrated strong potential as carriers for small-molecule drugs in PDC systems. Several polymer platforms, including PEG, PLGA, HPMA, and PGA, have been extensively investigated, and some have even progressed to clinical trials, such as HPMA–doxorubicin conjugates (PK1, PK2) and poly(L-glutamic acid)–paclitaxel (CT-2103, Xyotax) [45,54,76]. Despite these advances, many conventional polymer carriers still face limitations related to insufficient targeting, suboptimal therapeutic efficacy, and limited responsiveness to the complex biological environment. These challenges have increasingly shifted research efforts toward the development of functional polymers, which can provide additional capabilities such as stimuli-responsiveness or intrinsic biological activity, thereby enabling more precise and effective PDC-based drug delivery strategies.

## 4. Biofunctional Polymers

The previous chapter discussed biocompatible polymers, designed to remain inert in the body, avoiding the induction of undesired biological responses. This chapter introduces biofunctional polymers used as backbones for PDCs that actively interact with the biological environment to enhance drug delivery efficiency, while maintaining biocompatibility.

Biofunctional polymers are defined as a subset of polymers used in PDCs that, beyond serving as biocompatible carriers, possess functional properties that actively contribute to the therapeutic performance of the final PDCs. These polymers can interact with the biological environment or respond to specific physiological cues, thereby enabling controlled drug delivery and enhanced therapeutic efficacy. Accordingly, within the category of biofunctional polymers, we further classify them into two groups based on their mode of action in biological systems: stimuli-responsive polymers and bioactive polymers.

More specifically, stimuli-responsive polymers (also referred to as responsive or smart polymers) undergo physicochemical changes when exposed to specific environmental triggers such as variations in pH, temperature, enzymatic activity, or reactive oxygen species (ROS). These changes can promote site-specific or controlled drug release, cell and tissue targeting, or enhance cellular uptake of the PDC, particularly in pathological environments where such parameters are altered.

In contrast, bioactive polymers possess intrinsic biological activity and can directly interact with biological pathways, receptors, or cellular components. Rather than merely responding to environmental cues, these polymers can actively modulate the biological environment or influence the pharmacological activity of the conjugated drug. Thereby, they may contribute synergistically to the therapeutic outcome by promoting cellular uptake, targeting specific tissues, or inducing biological changes that enhance or alter drug efficacy.

First, polymers whose physicochemical properties change in response to biological stimuli and thereby improve delivery performance will be examined. Subsequently, polymers with intrinsic biological activity, capable of enhancing cell membrane permeability or exhibiting antibacterial or anticancer properties, will be discussed.

### 4.1. Stimuli-Responsive Polymers

The term stimuli-responsive polymer refers to a polymer able to undergo structural or chemical transformations solely when exposed to endogenous or exogenous stimuli such as light, temperature, electromagnetic field, pH, redox potential, or enzymes [20,125]. Traditional PDCs often exhibit suboptimal efficacy due to nonspecific or uncontrolled drug release. Incorporation of a stimuli-responsive linker enables precise spatiotemporal control of drug release, thereby enhancing therapeutic specificity and efficacy. Such responsiveness can be introduced through stimuli-responsive linkers (e.g., ester, hydrazone, or imine bonds) or through polymers designed to undergo controlled chemical or conformational changes in response to specific stimuli [126,127,128,129].

Here, we discuss most commonly applicable stimuli as triggers for stimuli-responsive PDCs, showing representative examples of applied polymers and how they influence the final properties of the PDC (Table 2).

#### 4.1.1. pH-Responsive Polymers

pH-responsive polymers exploit differences in acidity between physiological neutral pH (pH 7.4) and the pH of targeted physiological compartments, such as endosomes (6.5–4.5) or tumor tissues (6.5–7.0), to trigger controlled release of conjugated drugs [136,137]. These systems are particularly effective for targeting tumors or inflamed tissues, where acidic microenvironments facilitate polymer ionization, swelling, or cleavage of acid-labile linkers, ensuring localized and selective drug liberation. Representative examples include N-(2-hydroxypropyl) methacrylamide (HPMA)*,* chitosan, poly(amino acid), poly-histidine, poly-glutamic acid, poly-lysine, and poly(aspartic acid).

HPMA is a biocompatible, non-toxic synthetic polymer widely used in PDC formation due to the presence of an end-alcohol group readily available for conjugation with different drugs [138,139]. The drug conjugation often introduces a pH-responsive linker in the final PDC structure, enabling selective drug release in acidic microenvironments [140]. By using this strategy, pH-responsive targeting systems were developed, especially focusing on anticancer therapeutics, bringing forth the advantages of HPMA conjugated to an active targeting agent for an effective co-delivery of peptides and drugs like DOX or α-tocopheryl succinate [130,141,142,143,144].

Noack et al. developed a pH-responsive HPMA–DOX conjugate [145]. The star-shaped HPMA–DOX conjugate, in which DOX was linked via an acid-labile hydrazone bond, remained essentially stable at physiological pH (7.4) but underwent rapid drug release under acidic conditions (pH 5.5). This translated into a pH-dependent increase in cytotoxicity in vitro, with IC_50_ values showing an approximately 10-fold higher activity at pH 5.5 compared to pH 7.4 across all tested cancer cell lines. In xenograft models treated three times with a 2-fold DOX-equivalent dose of HPMA–DOX, complete tumor regression was achieved, while free DOX at standard dosing only induced partial tumor growth inhibition. Tumors characterized by a more acidic and hypoxic microenvironment (e.g., A2780cis) showed an immediate response due to a faster drug release, while less acidic tumors (1411HP) exhibited a delayed but ultimately strong response, consistent with intracellular rather than premature extracellular activation [145]. Overall, these results quantitatively underline how pH-responsiveness can amplify efficacy and enable superior in vivo antitumor activity at higher tolerated doses, emphasizing that controlled, acidity-triggered drug release is a key determinant in maximizing the therapeutic potential of HPMA-based PDCs.

Another promising approach is offered by the use of poly-glutamic acid (PGA) as a pH-responsive carrier for the delivery of anticancer drugs. PGA contains multiple carboxylic acid side chains, allowing facile conjugation of drugs via acid-labile linkers such as hydrazone bonds. Therefore, PGA is frequently used in cancer therapy for the conjugation of small drugs, including PTX and DOX [146]. A pH-responsive PGA–DOX conjugate was developed by Arroyo-Crespo et al. [131]. The system was synthesized by introducing a pH-labile hydrazone linker and aminoglutethimide (AGM) as a co-delivered agent. By analyzing different combinations of linker and drug loading, the study highlights how the introduction of the hydrazone linker led to a 10-fold higher release of the drug in an acidic environment (pH 5.0) compared to neutral pH (7.4). The release kinetics of DOX were directly connected to the biological and therapeutic output of the conjugate. A higher release of the drug at acidic pH led to enhanced efficacy of the conjugate when tested on 4T1 murine breast cancer cells. Additionally, in vivo studies showed a 50% tumor reduction with the conjugate compared to PBS-treated mice and demonstrated a less toxic administration profile of the PDC, as assessed by body weight and cardiotoxicity, compared to the free drug [131]. Ultimately, the strategic integration of pH-responsiveness into PDCs elevates the functionality of the carrier from passive transport to reactive, triggered delivery, representing a key advancement in the development of more precise and safer drug-delivery systems.

#### 4.1.2. Redox-Responsive Polymers

Beyond pH variations, intracellular redox potential is another critical physiological cue for selective, site-specific drug release. Redox-responsiveness exploits the reducing environment of the cytosol and the imbalance in oxidative and reductive species between healthy and diseased cells to enable site-specific drug release. Typically, intracellular compartments like the cytosol and cell nucleus present higher concentrations of reducing agents, such as glutathione (GSH), compared to the extracellular space (1–10 mM and 10–30 μM, respectively) [147,148]. By incorporating disulfide linkages, redox-sensitive PDCs achieve selective intracellular cleavage and efficient release of the therapeutic cargo. Thus, redox-responsive polymers often include poly(disulfide)s and disulfide-modified polymers like dextran disulfide derivatives, both of which degrade under reducing conditions, facilitating targeted release within tumor cells [149].

Another commonly employed functional group for imparting redox-responsiveness to polymers is boronates. Phenylboronic acid (PBA) is known for high sensitivity towards specific ROS, particularly ONOO^−^ and H_2_O_2_, which leads to oxidative cleavage of aryl–boron bonds through a well-established putative mechanism. Incorporation of PBA into polymer backbones enables both functional modification and ROS-triggered drug conjugation. In one example, PBA was used to conjugate the anti-inflammatory drug naproxen to dextran, resulting in a PDC, Nap-Dex [132]. The Nap-Dex exhibited excellent biocompatibility and completely released Nap within 20 min under 10 mM H_2_O_2_, confirming its ROS-responsive behavior. Notably, in LPS-stimulated macrophages, inflammation-associated ROS triggered drug release, leading to a marked reduction in the pro-inflammatory cytokines IL-6 and TNFα and restoring their levels to baseline. Given that Nap alone is known not to decrease TNFα, this enhanced anti-inflammatory effect is attributable to the ROS-responsive PBA linker, which scavenges ROS and thereby contributes additional therapeutic benefit [132].

Besides targeting inflammations, redox-responsive systems are widely used in tumor therapy. Xiao et al. developed a PDC based on poly(disulfide) (PSS) conjugated with DOX for the depletion of intracellular glutathione and drug release, exploiting the same triggering mechanism [133]. The PSS-DOX conjugate showed a selective increase in drug release when exposed to high GSH/reductive environments at the target. During this process, PSS–DOX significantly reduced intracellular GSH levels. In MCF-7 cells, it depleted GSH approximately two-fold more effectively than L-buthionine sulfoximine (BSO), a glutathione synthetase inhibitor used as a positive control. Resultingly, PSS–DOX achieved a tumor inhibition rate of up to 74% in vivo compared with control groups [133].

Collectively, these findings demonstrate that redox-responsive systems offer advantages beyond site-specific drug release. Considering that GSH and ROS function as key signaling mediators in the progression of inflammatory diseases and cancer, modulation of these biochemical factors can further enhance therapeutic outcomes. Thus, redox-responsive PDCs may not only enable controlled drug delivery but also potentiate the efficacy of the delivered agents by actively regulating the pathological microenvironment.

#### 4.1.3. Temperature-Responsive Polymers

In addition to pH and redox stimuli, temperature fluctuations offer an alternative and externally tunable mechanism to modulate polymer behavior and drug release. For example, malignant tissues show higher temperature compared to the surrounding healthy tissue due to angiogenesis and increased metabolic activity in the area. This increase in temperature (+0.2–0.4 °C) can be exploited in tumor treatment by using delivery systems able to release drug upon local temperature fluctuations [150,151]. Thermo-responsive systems exploit local or externally induced temperature changes to trigger reversible conformational transitions, enabling controlled modulation of drug release in stimuli-responsive PDCs. Many of these systems, such as poly(N-isopropylacrylamide) (PNIPAM) and poly(ethylene oxide)-poly(propylene oxide) block copolymers, exploit local thermal differences in inflamed or tumor tissues, or respond to externally applied heat [152,153]. Their tunable sol–gel or phase-transition behaviors enable high precision, making them suitable for injectable, thermally triggered, or externally controlled PDC systems.

LCST, Lower Critical Solution Temperature, is the temperature below which a polymer is soluble in a solvent and above which phase separation occurs due to reduced polymer–solvent interactions. This LCST is a key physicochemical parameter in the design of thermo-responsive PDCs. In the development of thermo-responsive systems, it is important to understand and investigate the molecular mechanisms underlying LCST-based thermo-responsive behavior. Stetsyshyn et al. provided useful insights on how dynamic hydrogen bonding and Van der Waals interactions drive temperature transitions in polymeric systems [154]. Such understanding is essential for predicting, controlling, and designing temperature-sensitive polymers for advanced applications.

Park et al. investigated the effects of monomer composition and drug conjugation degree on the LCST behavior of gradient copolymers composed of 2-n-propyl-2-oxazine (PropOzi) and 2-methoxycarbonylethyl-2-oxazoline (C2MestOx) by using cefazolin as conjugated drug [134]. PropOzi is relatively hydrophobic, whereas C2MestOx contains a more hydrophilic side chain. Accordingly, increasing the hydrophilic fraction of C2MestOx enhanced polymer–water interactions and resulted in an elevated LCST. In contrast, increasing the degree of cefazolin conjugation shifted the LCST to lower temperatures when hydrophobic contributions became dominant. Based on these findings, LCST was precisely tuned by controlling both polymer composition and the degree of drug conjugation. As a result, the polymer–cefazolin conjugate exhibited a phase transition near physiological temperature, with an LCST of 37 °C, promoting localized structural transformation and prolonged drug retention with sustained release. Consequently, it demonstrated effective antibacterial activity in a time-kill assay against *E. coli* [134]. Thermo-responsive systems are closely associated with LCST-based solubility control, and temperature is also directly linked to the reactivity of chemically driven stimuli-responsive systems discussed earlier. When combined, these mechanisms enable simultaneous regulation of phase transition and reaction kinetics, providing functional advantages. Farjadian et al. developed a temperature and pH-responsive PDC by combining a random copolymer of PNIPAM and polyacrylamide (poly(NIPAM-co-AAm)) conjugated with DOX by a Schiff-base linkage [135]. After determining the LCST of the final conjugate to be at 40 °C, a temperature- and pH-dependent release of DOX was performed by treating the polymer at pH 5 and 7.4, as well as at 37 and 42 °C (below and above the LCST). By comparing DOX release at pH 5, which was proven to be higher than pH 7.4, between the samples treated at 37 and 42 °C, a 2.1-fold higher release of the drug was observed. Finally, in vitro studies confirmed the efficacy of the system on cancer cells with a cytotoxic effect comparable to that of free DOX on MCF-7 cells, indicating the promising application of poly(NIPAM-co-AAm)-DOX as a temperature and pH-triggered delivery system for targeted cancer therapy [135].

In summary, reactive stimuli-responsive polymers transform PDCs into smart delivery systems that precisely release therapeutic payloads in response to disease-specific triggers, thereby enhancing efficacy and reducing unwanted toxicity. In addition to the representative systems discussed above, other external and internal stimuli, such as light, ultrasound, magnetic fields, and enzymatic activity, have also been employed to regulate drug conjugation and release behavior [155,156,157,158].

The choice of stimulus in PDCs is closely linked to the intended therapeutic application. pH-responsive polymers are predominantly employed for tumor and inflammation-targeted therapies, exploiting acidic microenvironments to trigger selective drug release. Redox-responsive systems, including disulfide- and boronate-containing polymers, are primarily used for intracellular delivery and modulation of oxidative stress, enabling site-specific activation in cancer or inflamed tissues. Temperature-responsive polymers, often designed around LCST transitions, provide externally or locally tunable release, making them suitable for controlled therapy in regions with mild hyperthermia or externally applied heat. In all cases, the stimuli-responsive polymer acts as more than a passive carrier: it senses environmental cues and actively regulates drug release, enhancing selectivity, efficacy, and safety by minimizing off-target exposure. Thereby, using stimuli-responsive, biofunctional polymers for drug conjugation, the resulting PDCs represent advanced drug-delivery systems compared to regular, non-responsive conjugates.

Based on this responsive functionality, the next section will focus on biologically active polymers, possessing the ability to engage in therapeutic interactions and contribute to therapeutic efficacy.

### 4.2. Biologically Active Polymers

Biologically active polymers play a revolutionary role in PDCs by offering more than just structural support. These polymers possess inherent biological functions, such as receptor recognition [159], cell adhesion [160], or therapeutic activity [161], that can actively contribute to the overall efficacy of the drug delivery system. By engaging directly with biological targets or modulating the local microenvironment, they can enhance drug accumulation at the delivery site, improve cellular uptake, or even provide synergistic effects with the conjugated drug. This dual functionality, contributed from both carrier and active compound, enables advanced PDCs, where multifunctionality and specificity are essential.

#### 4.2.1. Gelatin

Gelatin is a natural, biodegradable polymer that derives from hydrolytic degradation of collagen, showing excellent biocompatibility and low immunogenicity [162,163]. As a protein, it is composed of amino acids linked via peptide bonds, among which proline, hydroxyproline, and glycine represent the majority of the contained amino acids [164]. There are multiple collagen sources used for the production of gelatin, from which porcine and bovine sources are the most frequently utilized origins [165]. Nevertheless, alternative sources for gelatin, such as fish gelatin or biotechnologically produced gelatin, have been explored, offering a variety of amino acid compositions and, therefore, interesting opportunities for chemical modification and crosslinking [166,167]. One major advantage of gelatin as a compound in drug delivery systems derives from its biodegradability, mainly driven by enzymatic degradation through proteases such as trypsin, pepsin, and aminopeptidase N, native to the human body [168]. This intrinsic biodegradability not only facilitates drug release from encapsulated or conjugated active pharmaceutical ingredients but also leads to biologically active hydrolysates, which can be beneficial during therapy [169,170,171].

The proteinaceous character of gelatin, combined with its enzymatic degradability and favorable biocompatibility, makes it a suitable and well-defined drug delivery system for small molecules as well as macromolecular drugs [172,173]. In addition to its application as a carrier matrix for encapsulated drugs, gelatin can be used to create PDCs.

In a study published by Ofner et al., the anti-cancer drug methotrexate (MTX) was conjugated to gelatin, which was previously modified with citraconic anhydride [174]. After modification, the MTX was bound to the gelatin’s carboxylic groups via EDC coupling. The conjugate demonstrated a significant uptake into tumor cells while releasing the MTX both via hydrolysis and enzymatic degradation using the protease cathepsin B. The degradation of the PDC, resulting in free MTX and gelatin hydrolysates, bears substantial potential for cancer treatment, as these hydrolysates can possess antioxidative properties [171,175]. It could be shown that hydrolysates from various gelatin sources have significant radical-scavenging activity after enzymatic degradation by different native proteases [175,176]. The dualism of gelatin hydrolysates and MTX offered by the degradation of the presented gelatin PDC can, therefore, be viewed as a combined treatment due to its antioxidative properties, which have been proven to play a supportive role in anti-cancer therapy [177].

A different study presented the conjugation of gelatin from bovine and porcine origins to the antituberculosis drug isoniazid [178]. The coupling was prepared by the addition of thionyl chloride to carboxypolystyrene resin in N-methyl pyrrolidone, resulting in the chlorination of the C-terminal groups of the resin. Through the addition of gelatin, an amide bond between the biopolymer and the resin was formed, catalyzed by pyridine. After introducing acyl chloride groups to the gelatin via thionyl chloride, the isoniazid’s hydrazinium groups were conjugated to these acyl chloride groups, forming an amide bond, resulting in a stable PDC [178,179]. The synthesized conjugate displayed excellent antimycobacterial activity while simultaneously reducing the number of toxic metabolites compared to the free drug. However, during tuberculosis therapy with isoniazid, increased blood pressure levels and hypertension are common side effects, especially in vulnerable patient groups [180,181]. Hydrolysates derived from bovine and marine gelatin sources through enzymatic degradation expressed significant antihypertensive properties by angiotensin-converting enzyme (ACE) inhibition [182,183,184]. This antihypertensive activity of gelatin can be of beneficial use during the treatment of tuberculosis patients with isoniazid, suffering from drug-induced raised blood pressure levels. While the aforementioned PDC undergoes degradation by proteases, free isoniazid gets released from the conjugate by cleavage of the amide bonds, whereas simultaneously gelatin hydrolysates originate during this process, reducing hypertensive side effects from the drug.

Both examples highlight the advantageous use of PDCs composed of the bioactive polymer gelatin, either used as a supplementary therapy, for instance, during cancer therapy, or as a smart way of countering adverse effects from conjugated drugs and, therefore, ease pharmacological treatment for patients. However, some limitations for gelatin-based PDCs remain in the form of batch-to-batch variety due to the animal-derived origin of the biopolymer, as well as rather low therapeutically efficacious concentrations of gelatin hydrolysates [185]. Regarding the first problem, biotechnologically produced gelatin can present a more reliable source, leading to gelatin with very high purity and consistency and circumventing ethical concerns [186]. The challenge of low hydrolysate concentrations needs to be further evaluated in in vivo experiments to determine clinically relevant concentrations and dosages. Further, additional studies investigating the biological activity of biotechnologically produced gelatin and of the resulting PDCs should be carried out in the future.

**Figure 4:**
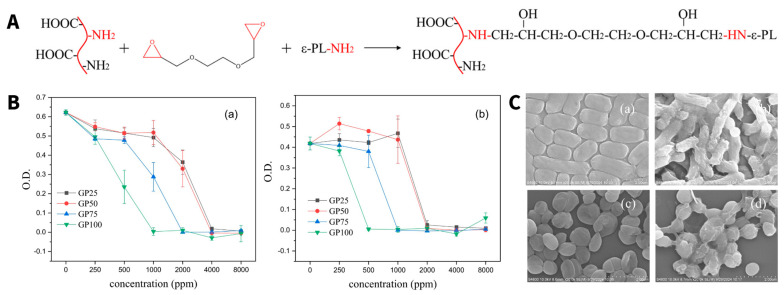
ɛ-PL conjugation on gelatin via ring-opening addition with ethylene glycol diglycidyl ether as crosslinker (**A**). Antibacterial effect of gelatin–poly-lysine conjugates on (**a**) *E. coli* and (**b**) *S. aureus* (**B**). SEM images of *E. coli* (**a**) and *S. aureus* (**c**) membrane morphology before antibacterial treatments, and *E. coli* (**b**) and *S. aureus* (**d**) after treatment with GP25 (**C**). Reproduced with permission from Cao et al., *Journal of Applied Polymer Science* [187]; copyright Wiley 2025.

Additionally, gelatin can be used as a compound in the synthesis of a novel bioactive polymer when conjugated to the polypeptide ɛ-poly-lysine (ɛ-PL) [187]. In this study, gelatin was covalently conjugated to ɛ-PL via ring-opening addition using ethylene glycol diglycidyl ether as a linker (Figure 4A). As we will discuss in the next paragraph, ɛ-PL possesses intrinsic antibacterial activity against multiple bacterial strains, i.e., *S. aureus* [188]. The resulting polymer displays significant antibacterial efficacy against *E. coli* and *S. aureus* with a clear dependency on the amount of conjugated ɛ-PL (Figure 4B,C) [187]. This example showcases the possibility of combining two bioactive polymers into a novel polymer conjugate, offering new opportunities for its use as a compound in PDCs. The properties and the use of poly-L-lysine (PL) as a stand-alone polymer in PDC formation will be further discussed in the next section.

#### 4.2.2. Poly-lysine

Poly-L-lysine (PL) is a cationic homopolypeptide composed of L-lysine residues, which are connected via peptide bonds between the ɑ- or ɛ-amino group and ɑ-carboxyl groups of the monomers, to form ɑ-PL and ɛ-PL, respectively. While ɑ-PL is a synthetic product of a polycondensation reaction, ɛ-PL is naturally produced by *Streptomyces albulus* and approved by the FDA as a food preservative [189,190]. Both polymers display high water solubility, thermal stability, along with biocompatibility and biodegradability, supporting their suitability for biomedical applications [191,192]. ɑ-PL is widely used as a nanocarrier due to its numerous amino groups available for modification and drug conjugation in addition to its positive charge, which is investigated as a bacterial membrane-disrupting agent [189]. However, ɑ-PL’s positive charges are not only responsible for its antibacterial activity, but also for its cytotoxicity. This cytotoxicity appears to be associated with the molecular weight of the polymer, resulting in higher toxicity for high-MW ɑ-PL [193,194]. While cytotoxicity serves as a limitation of ɑ-PL, ɛ-PL has emerged as a bioactive polymer widely used for diverse applications, from cancer treatment to antibacterial, antifungal, and antioxidative therapies, taking advantage of its chemotherapeutic properties [195,196]. The dual character of ɛ-PL, as a therapeutic agent and carrier, is an intriguing starting point for a new class of PDCs, combining great polymeric carrier properties with intrinsic bioactivity, where the polymer plays an active role in the delivery system to have a conjugate completely composed of active compounds.

**Figure 5:**
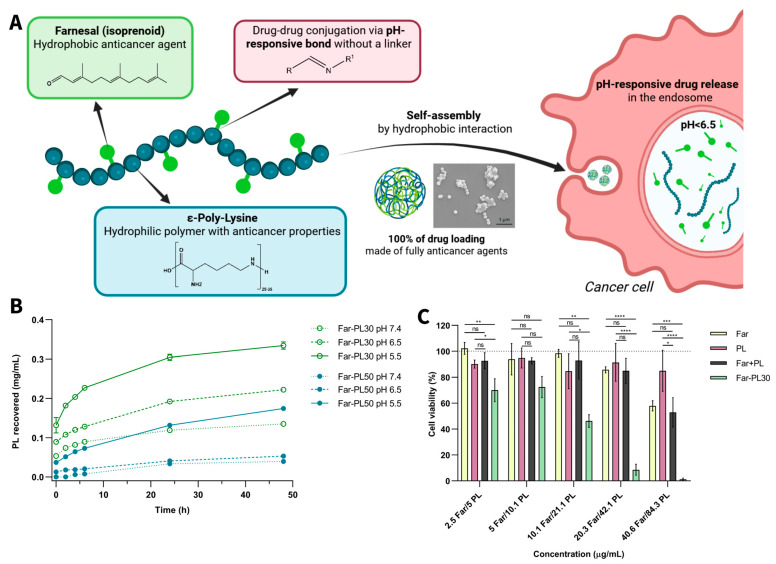
Schematic illustration of Far-PL conjugates (**A**). pH-dependent release of PL from Far-PL30 and Far-PL50 under neutral and acidic conditions (**B**). Far-PL30 anticancer activity on A549 cells, compared to free Far, free PL, and their physical mixture. * *p*  < 0.05, ** *p*  < 0.01, *** *p* < 0.001, **** *p*  < 0.0001, ns, nonsignificant. (**C**). Reproduced with permission from Passi et al., *ChemMedChem* [198]; copyright Wiley 2026.

Yu et al. investigated the effect of α-Poly-lysine (α-PL) and ε-Poly-lysine (ε-PL) as carriers for the delivery of methotrexate (MTX) [197]. Both polymers were used to form nanoparticles loaded with MTX (α-PL/MTX NPs, ε-PL/MTX NPs), resulting in smaller particles for ε-PL compared to α-PL (115 and 185 nm, respectively). Nanoparticles showed comparable polydispersity indices (PDIs), zeta-potential, and drug loading. The main difference in the choice of the carrier was proven by observing the final nanoparticles with a scanning electron microscope, showing a spiral/rod-shaped structure for ε-PL, while α-PL resulted in agglomerated structures. This can be explained by the different lengths of the side chains, resulting in different interactions and assembly processes. When tested in vitro on 4T1 cells, both formulations showed higher efficacy than MTX alone. Notably, ε-PL/MTX NPs exhibited 2.6-fold higher efficacy than α-PL/MTX NPs in vitro and approximately 1.27-fold greater antitumor efficacy. In vivo testing on 4T1 tumor-bearing mice [197]. The study discusses the superior activity of ε-PL/MTX being attributed to the different morphology of the particles and possible synergy between ε-PL and MTX. Based on the established anticancer activity of ε-PL, this study demonstrates its capacity to enhance therapeutic outcomes through synergy with the delivered drug. Moreover, ɛ-PL-based systems can be engineered to exhibit pH- or enzyme-responsive behavior through cleavable bonds or tailored side-chain modifications. Our recent study demonstrated this concept by employing ɛ-PL as an active carrier for the hydrophobic anticancer isoprenoid farnesal (Far) [198]. The degree of Far conjugation allowed modulation of PDC hydrophobicity and acid-triggered drug release kinetics, with lower Far content (Far-PL30) leading to more rapid release than Far-PL50 under acidic conditions (Figure 5). When tested on A549 lung cancer cells, Far-PL demonstrated notably improved efficacy compared to free ɛ-PL or Far alone. In particular, Far-PL30 exhibited the most pronounced anticancer activity, inducing near-complete loss of cell viability under conditions (125 µg/mL) where neither Far nor ɛ-PL alone, nor their physical mixture, produced comparable effects. These findings highlight the critical role of covalent conjugation for enhanced anticancer performance. The increased efficacy can be connected to multiple synergistic factors: improved solubility and intracellular delivery of Far, enhanced internalization of ɛ-PL through amphiphilicity, and acid-triggered activation within endosomes. This was further confirmed by inhibition studies with chloroquine, which significantly reduced activity by blocking endosomal acidification. The results of the chloroquine study confirmed that cellular uptake and pH-responsive intracellular drug release within endosomal compartments are critical determinants of therapeutic efficacy. More importantly, Far-PL displayed selective activity toward cancerous A549 cells while inducing considerably lower toxicity in non-cancerous Arlo lung epithelial cells. Beyond biological activity, the amphiphilic conjugates self-assembled into stable nanoparticles entirely composed of active compounds, enabling 100% drug content, significantly surpassing conventional nanoparticles, which typically load only 5–10 wt% of drug [198]. This approach demonstrates how pairing two bioactive components through a stimuli-responsive linkage can overcome solubility limitations, enhance intracellular targeting, and greatly improve therapeutic potential.

In summary, poly-lysine represents a versatile class of carriers whose role in PDCs extends far beyond simple drug delivery. Even though both α- and ε-PL offer highly functionalizable backbones for conjugation, ε-PL distinguishes itself as a naturally derived polymer with intrinsic antimicrobial, antioxidant, and anticancer properties. This inherent bioactivity allows PL-based systems to act synergistically with their cargo, enhancing therapeutic outcomes through both improved intracellular delivery and cooperative biological mechanisms. Moreover, their tunable charge, degradability, and capacity to form responsive architectures enable precise control over drug release and cellular interactions [199,200]. In particular, ε-PL offers a unique advantage as a naturally derived polymer with antimicrobial and anticancer properties, enabling synergistic therapeutic effects when combined with conjugated drugs. Nevertheless, despite its regulatory approval as a food preservative and a favorable biocompatibility profile, ε-PL remains underexplored in clinical drug delivery applications, and further studies are required to fully evaluate its pharmacokinetics, long-term safety, and in vivo efficacy. Continued optimization of polymer architecture, molecular weight, and charge shielding strategies may help overcome current limitations, potentially establishing poly-lysine-based systems as multifunctional platforms for next-generation PDCs.

#### 4.2.3. Hyaluronic Acid

**Figure 6:**
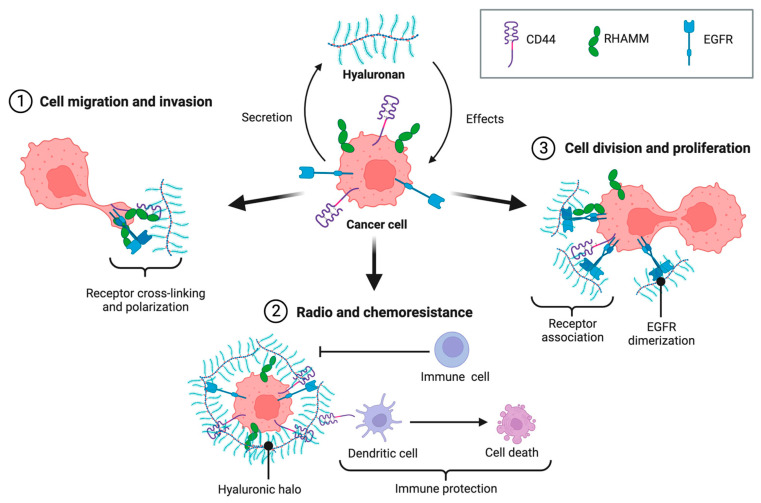
Hyaluronan impact on cancer cell behavior. By interacting with specific receptors on the cell, including CD44, RHAMM, and EGFR, hyaluronan can influence (1) cell migration and invasion, (2) regulate radio and chemoresistance, and (3) interfere with cell division and proliferation. Reproduced with permission from Cirillo, *International Journal of Molecular Sciences* [206]; copyright MDPI 2023.

Hyaluronic acid (HA) is a naturally occurring glycosaminoglycan, composed of repeating disaccharide units of glucuronic acid and N-acetylglucosamine. It is universally distributed in the extracellular matrix and plays a key role in tissue hydration, lubrication and cell signaling [201]. In addition to its biocompatibility and biodegradability, HA is frequently used for biomedical applications due to its targeting properties [202]. HA is one of the main ligands for CD44, a family of glycoproteins expressed in various cell types but overexpressed in cancer cells (Figure 6) [203,204]. More specifically, an important isoform that showed high binding affinity for HA is CD44v, a marker for cancer stem cells. The protein can be found in a small population of cells within tumors and is responsible for the self-renewal capacity of tumor mass, in addition to the ability to resist conventional therapies [205]. Hence, the ability of HA to target those receptors is highly useful in the field of drug delivery and PDC development, labeling HA as a promising carrier with good biocompatibility, biodegradability, and low immunogenicity, but also active targeting abilities to safely deliver the conjugated drug to cancer cells [206].

In the context of PDCs, HA is particularly valuable due to its ability to bind to specific cell surface receptors, such as the previously mentioned CD44, which are overexpressed in various tumors and inflamed tissue [207]. This endogenous targeting ability enables selective accumulation of HA-based PDCs at disease sites, enhancing therapeutic efficacy while minimizing systemic toxicity. Additionally, HA’s hydrophilic nature improves the solubility of hydrophobic drugs, and its multiple functional groups allow for sufficient chemical conjugation [208]. These features make HA an attractive polymer for developing receptor-targeted, biologically active drug conjugates.

Thummarati et al. developed a dual-delivery system based on hyaluronic acid for the targeted administration of gemcitabine (GEM) and curcumin (CUR). In their work, GEM was conjugated with CUR-HA (ChH) via two different chemical mechanisms, generating either ester-linked random grafts (GeChH) or hydrazone-linked block grafts ((GhC)hH), leading to both conjugates self-assembling into nanoparticles [209]. Drug release studies demonstrated the pH-responsive behavior of both systems, with accelerated GEM and CUR release under acidic conditions (pH 5 and 6.5) and slower release at physiological pH (pH 7.4). GeChH nanoparticles showed a faster release rate compared to (GhC)hH nanoparticles, with a 1.2-fold increase in the cumulative amount of GEM released at pH 6.5 after 24 h. Notably, the (GhC)hH nanoparticles displayed superior control over GEM release at pH 7.4, with a 1.4-fold lower release of the drug due to the greater stability of hydrazone linkages relative to ester bonds. When tested on multiple cancer cell lines (PANC-1, A549, HCT116, Caco-2), both nanoparticle formulations outperformed free GEM, free CUR, and the physical mixture of the drugs, exhibiting enhanced cytotoxicity and synergistic activity. The (GhC)hH nanoparticles proved to be more effective again, displaying lower IC_50_ values and stronger synergy, especially in fast-growing and CD44-overexpressing cells (A549, HCT116). Cellular uptake studies confirmed that the two systems were internalized efficiently via HA-CD44 interactions, with the block-graft nanoparticles demonstrating higher uptake due to their smaller size and more favorable charge (221 nm and +25 mV for (GhC)hH compared to 258 nm and +9 mV for GeChH) [209]. Overall, the work illustrated the effective use of HA as a targeting agent, able to make an impact on the cellular uptake and biological performance of HA-based PDCs.

Similarly, Yin et al. designed a redox-responsive hyaluronic acid–paclitaxel conjugate (HA-ss-PTX) to enhance intracellular delivery and tumor-specific activation of the drug [210]. In this system, PTX was covalently attached to HA of different molecular weights (9.5, 35, and 770 kDa) through a disulfide linker, enabling the conjugates to form micelles. Compared to PEG-based analogues (mPEG-ss-PTX), HA-ss-PTX displayed superior drug-loading capacity (7.2 and 26.7%, respectively) and effective PTX release under reductive conditions, with a 1.1-fold higher release of PTX after 96 h. Coumarin-6 (C6) was used as a fluorescent probe to investigate the cellular internalization and intracellular release from the micelles. All HA-ss-PTX conjugates showed enhanced cellular internalization, attributed to the CD44-mediated uptake. Among the tested formulations, HA9.5-ss-PTX exhibited the most favorable characteristics, including efficient endocytosis via CD44–caveolae pathways and potent intracellular PTX release triggered by glutathione. In vivo, HA9.5-ss-PTX achieved significantly improved antitumor efficacy, with a tumor inhibition rate of 83.3%, outperforming both mPEG-ss-PTX (51.4%) and Taxol [210]. The enhanced performance of HA-ss-PTX originated from the synergistic combination of HA’s intrinsic CD44-targeting ability, the redox-cleavable linker enabling selective intracellular activation, and the optimized HA molecular weight, confirming HA-ss-PTX as a highly promising platform for controlled, tumor-specific chemotherapy.

Overall, the use of hyaluronic acid in PDCs highlights its dual role as a biocompatible carrier as well as a biologically active component that actively contributes to therapeutic performance. Its natural affinity for CD44 and related receptors enables intrinsic targeting of cancer cells, allowing HA-based systems to localize more efficiently at disease sites while reducing off-target effects. Beyond passive delivery, HA’s biological interactions enhance cellular internalization pathways and support more effective drug action, underscoring its value as an active participant in treatment rather than a neutral scaffold. However, despite these promising features, translation to clinical applications remains challenging. Variability in CD44 expression among patients, potential enzymatic degradation in vivo, and scale-up of reproducible conjugation strategies are key hurdles. Moreover, long-term safety, immunogenicity upon repeated administration, and pharmacokinetic behavior require thorough investigation. Addressing these issues is essential to fully realize HA-based PDCs as effective, targeted therapies.

#### 4.2.4. Chitosan

The bioactive polymer chitosan is derived from chitin after deacetylation and consists of the two amino sugars glucosamine and *N*-acetyl-glucosamine, linked via b-1,4-glycosidic bonds [211]. Crustaceans, e.g., shrimp or crabs, are the most common source for chitosan extraction, whereas fungi represent a more recent origin for the raw material, which is still undergoing extensive research [211,212]. The natural origin of chitosan as a polysaccharide in combination with its lysozyme-cleavable glycosidic bonds attributes the copolymer a good biocompatibility as well as sufficient biodegradability [213,214]. Chitosan is generally determined by its degree of deacetylation (DD), which also provides the foundation of differentiation between chitin and its derivative chitosan, with the latter showing a usual DD of 75–85% [215]. The DD has a crucial impact on the solubility of the polymer, with mostly deacetylated chitosan showing limited water solubility at acidic pH and no solubility at pH values over 7. In contrast, chitosan with a low DD displays full water solubility at all pH conditions [216]. However, a partial deacetylation reaction leaves the polymer with a high number of primary amino groups suitable for chemical modification, including the conjugation of various drugs to the polymer to synthesize PDCs [217]. It is therefore important to identify a compromise for the DD, balancing water solubility and the number of accessible groups for drug conjugation.

In addition to their chemical accessibility, the primary amino groups of chitosan are prone to protonation, rendering the polymer positively charged at lower pH values [218]. Due to its cationic character, chitosan possesses an intrinsic antimicrobial activity against various bacteria and fungi, with a minimum growth inhibitory concentration (MIC) of 20 ppm against *S. aureus* and *E. coli* [77,219]. Considering the polymer’s antibacterial activity, the synthesis of chitosan–antibiotic conjugates represents a promising approach for boosting the efficacy of antibiotics. This principle was used in a study by Zhang et al. in which the aminoglycoside antibiotic streptomycin was conjugated to chitosan via the reduction in the Schiff base originating from primary amino groups of the polymer and their linkage with aldehyde groups from the antibiotic [220]. The resulting PDC was used to form a complex with human serum albumin, lowering the drug’s cytotoxicity as well as facilitating its intracellular uptake. The conjugate showed an improved efficacy against bacteria compared to chitosan and streptomycin alone, proving its superiority in the treatment of intracellular bacteria in vivo [221]. The study highlights the antibiotic synergy between chitosan, a natural antimicrobial agent, and streptomycin, making it an advantageous system for the treatment of bacterial infections.

**Figure 7:**
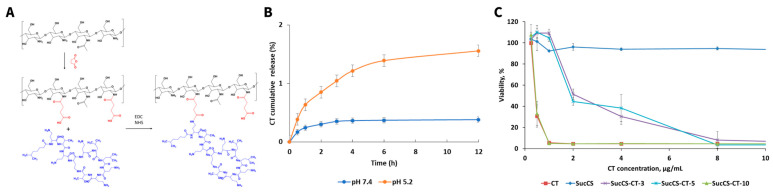
Synthesis of the succinyl chitosan–colistin conjugate (SucCS-CT) (**A**). CT releases SucCS-CT-10 at pH 7.4 and 5.2 at 37 °C over 12 h. (**B**). Antibacterial activity of the conjugates against *P. aeruginosa* (**C**). Reproduced with permission from Dubashynskaya et al., *International Journal of Molecular Sciences* [222]; copyright MDPI 2023.

In a different study, chitosan was chemically modified using succinyl anhydride and subsequently conjugated with the glycopeptide antibiotic colistin (Figure 7) [222]. Succinyl anhydride introduced carboxyl groups to the chitosan, which were, in the next step, linked to the peptide antibiotic’s amine groups using EDC as a crosslinking reagent. The synthesized PDC displayed a conjugation efficiency between 54 and 100% and a degree of substitution with colistin of 3–8%. Colistin is a naturally produced antimicrobial agent that is highly effective against Gram-negative bacteria [223]. However, the antibiotic is known for a distinct nephrotoxicity, limiting its application in vulnerable patient groups [224]. By conjugating the glycopeptide to succinyl chitosan, the nephrotoxic effects of colistin could be reduced by 20 to 60% while maintaining a strong antibiotic efficacy [222]. Succinyl chitosan proved to be effective against both Gram-positive and Gram-negative bacteria, making it a suitable conjugation partner for antibiotic PDCs [225]. In another study, chemical modification of chitosan was carried out to enable drug conjugation, whereby the conjugated drug, tobramycin, functioned as a crosslinker within chitosan-based gene delivery carriers [226]. This strategy leverages the intrinsic physicochemical properties and biological activity of chitosan while enhancing structural stability through drug-mediated crosslinking.

In conclusion, chitosan represents a highly attractive bioactive polymer for PDC formation due to its intrinsic mucoadhesive and cationic nature, which enhances interaction with negatively charged biological membranes and improves drug absorption and bioavailability. Nevertheless, several limitations hinder its broader application. Most notably, chitosan exhibits poor solubility at physiological pH, being primarily soluble only under acidic conditions, which restricts its use in systemic delivery [227]. Stability issues, including hydrolytic degradation and swelling in aqueous environments, can lead to premature drug release and reduced shelf life [228,229]. These challenges often necessitate chemical modification or formulation strategies to optimize their performance in PDC systems. Consequently, the influence of these chemical modifications on chitosan’s intrinsic bioactivity needs to be assessed in more detail in future studies.

#### 4.2.5. Biodynamers

**Figure 8:**
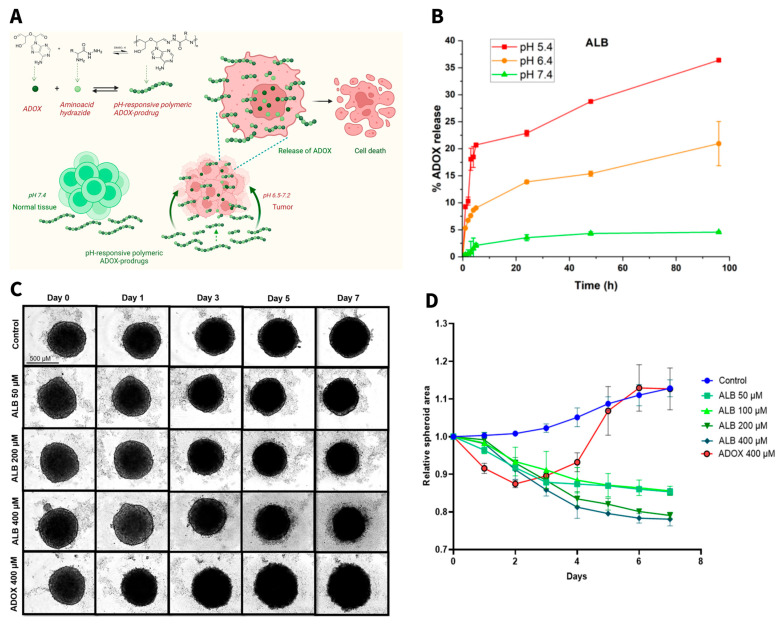
Schematic illustration of ADOX biodynamers; the conjugation of ADOX with amino acid hydrazide results in the formation of a pH-responsive PDC for the safe delivery of ADOX selectively to cancer tissues (**A**). Release profile of ADOX from ALB (**B**). Images of spheroids were captured under a microscope at different time points after treatment with ALB and ADOX. Scale bar: 500 µm (**C**). The relative spheroid area of HCT116 spheroids was analyzed from the images of (**C**) (n = 3, N = 3) (**D**). Reproduced with permission from Nguyen et al., *Journal of Controlled Release* [236]; copyright Elsevier 2025.

Biodynamers (BDs) represent a distinctive class of dynamic polymers constructed through reversible covalent chemistry, most commonly via hydrazone or imine linkages between multifunctional aldehydes and amino acid-derived hydrazides [230,231]. They possess the unique ability to reversibly polymerize, depolymerize, and reorganize their structure in response to environmental stimuli such as pH, ionic strength, or biomolecular interactions [232,233,234]. BDs have been explored as carriers for nucleic acids and proteins, and recently have been established as a polymeric backbone in PDC formation for the delivery of small molecules [226,235]. Nguyen et al. developed a novel ADOX-Lys biodynamer by conjugating adenosine dialdehyde (ADOX) to the already established lysine biodynamer [236,237]. ADOX is an adenosine analogue that can be used in cancer therapy as an indirect methylation inhibitor, but its clinical use is limited by its untargeted toxicity [238]. By conjugating ADOX to Lys-biodynamer, it was possible to achieve a system stable at physiological pH, and able to selectively release the drug once the acidic tumor environment is reached (Figure 8). When tested on human cancer cells (HCT116, MCF-7, and SW480), the ADOX-Lys BD showed negligible cytotoxicity at neutral pH, while under acidic conditions, up to 60% of the activity of free ADOX was recovered. Furthermore, testing the system on HCT116 spheroids led to a 40% reduction in the spheroids’ size after 7 days of treatment. The conjugation of ADOX with Lys-biodynamer resulted in enhanced cellular uptake under simulated acidic tumor microenvironment, allowing selective delivery of the drug to cancer cells and effective penetration into the tumor tissue [236].

Next to their role as carriers, biodynamers can possess intrinsic biological activity, which can synergize with or even amplify the effect of the delivered therapeutic. This dual function has been investigated in cancer therapy and anti-infective strategies.

BDs have emerged as powerful adjuvants in antibacterial therapy, where their intrinsic activity is exploited by potentiating the efficacy of conventional antibiotics. A notable example is provided by arginine-based biodynamers (Arg-BDs) designed to target Gram-negative bacteria [239]. While these Arg-BDs displayed antibacterial activity comparable to poly-L-arginine but with drastically reduced mammalian cytotoxicity, their most striking effect was observed in combination therapy. When co-administered with colistin, the compound reduced the minimum inhibitory concentration of the antibiotic against *E. coli* by up to 32-fold, effectively acting as an antibiotic potentiator that enhances membrane permeability and bacterial drug uptake without increasing host toxicity [239].

In summary, these examples highlight how BDs transcend the traditional role of polymeric carriers, functioning instead as active components that combine dynamic structural adaptability with inherent bioactivity, thereby enabling synergistic therapeutic effects. This dual functionality supports applications in both cancer and infectious disease treatment, providing selective delivery, improved cellular uptake, and potentiation of conventional drugs. Nevertheless, translation to clinical use faces challenges, including ensuring reproducibility of dynamic polymer structures at scale, predictable pharmacokinetics, long-term stability in complex biological fluids, and minimizing potential off-target effects. Addressing these factors is essential to harness the full potential of biodynamers as clinically viable, active polymer–drug conjugates.

Taken together, the five classes of bioactive polymers discussed demonstrate that the polymer component in PDCs can actively dictate therapeutic performance rather than acting as a passive carrier. Each system contributes through a distinct biological mechanism that directly impacts targeting, cellular uptake, and efficacy. Hyaluronic acid represents the most explicit example of receptor-mediated targeting: through CD44 binding, HA conjugation has been shown to significantly enhance tumor cell internalization compared to free drugs. This active targeting translates into substantially improved in vivo efficacy, which is also reflected in higher tumor inhibition rates compared to non-targeted systems. In contrast, cationic polymers such as poly-lysine and chitosan primarily enhance uptake via electrostatic interactions with negatively charged cell membranes, promoting adsorptive endocytosis and membrane destabilization. This mechanism can lead to multi-fold increases in intracellular delivery and therapeutic efficacy, as observed for ε-PL systems and chitosan–antibiotic conjugates that outperform both free drug and polymer alone through synergistic antimicrobial activity.

Gelatin modulates PDC performance predominantly through its enzymatic degradability: protease-triggered breakdown not only enables controlled drug release but also generates bioactive hydrolysates that contribute secondary therapeutic effects (e.g., antioxidant or antihypertensive activity), thereby functionally augmenting the drug payload and mitigating side effects. Finally, biodynamers introduce a dynamic and stimuli-responsive dimension, where reversible covalent bonds enable environment-specific activation, and their intrinsic bioactivity can potentiate co-administered drugs. Collectively, these examples establish that biofunctional polymers enhance PDC performance through three principal mechanisms: active targeting and receptor-mediated uptake (hyaluronic acid), membrane interaction-driven internalization and intracellular delivery enhancement (poly-lysine, chitosan), and bioactivity-coupled release and therapeutic synergy (gelatin, biodynamers). Compared to conventional inert carriers, these systems consistently yield higher cellular uptake, improved intracellular drug activation, and synergistic therapeutic effects, ultimately transforming PDCs into integrated therapeutic platforms in which both the polymer and the drug contribute to the overall efficacy.

## 5. Perspective and Conclusions

In this review, we discussed that the selection of the polymer is critical for ensuring safety and enabling the stable delivery of the conjugated drug in PDCs. Appropriately engineered polymers could strategically regulate drug release under physiological conditions and enhance targeting efficiency. Moreover, the use of biofunctional polymers offered the potential to actively modulate the biological environment or augment the therapeutic efficacy of the payload. From this perspective, polymers should be viewed not simply as passive carriers that control the properties of conjugated drugs, but as essential design elements that enable more advanced PDC functionalities.

However, when using the polymers as active carriers, the following factors should be carefully considered to maximize their potential and their translational success. Natural polymers such as poly-lysine, hyaluronic acid, chitosan, gelatin, or polysaccharides combine biocompatibility, biodegradability, and intrinsic bioactivity, but they often suffer from structural heterogeneity. This is because natural sources generally provide polymer chains of varying length, purity, and composition, which complicates reproducibility and precise control of the physicochemical properties required for pharmaceutical applications [240,241]. This variability, together with the presence of impurities inherent to biological sources, can lead to inconsistent solubility and degradation profiles, ultimately affecting formulation stability and drug-release behavior [241].

Looking forward, future research should prioritize the rational design and standardization of natural bioactive PDCs. One promising strategy is to develop recombinant, chemically defined versions of natural polymers (for example, by biosynthesis or enzymatic modification) to reduce heterogeneity and improve reproducibility. Advances in controlled polymerization and site-specific conjugation could help produce well-defined, uniform conjugates.

Meanwhile, synthetic biofunctional polymers produced through well-controlled polymerization techniques experience fewer challenges related to the structural heterogeneity and batch-to-batch variability often associated with natural polymers. However, unlike long-established natural polymers with extensive clinical experience and well-documented safety profiles, recently developed synthetic biofunctional polymers lack comprehensive data regarding their long-term biological impact and secondary physiological activities. PEG, for example, has been widely adopted due to its stealth properties and other advantages, yet repeated administration has been reported to induce anti-PEG antibody formation, accelerated blood clearance, hypersensitivity, and allergic reactions, highlighting emerging concerns regarding immunogenicity. Consequently, synthetic biofunctional polymers and their degradation products require rigorous evaluation of their physiological impact, particularly under long-term dosing conditions.

In particular, systematic studies are required to elucidate biodistribution, immunogenicity, long-term degradation behavior, and overall safety. Computational and machine-learning-based approaches, which have become essential tools across recent research, can play a crucial role by enabling correlation analysis among polymer structure, conjugation chemistry, and in vivo performance, thereby supporting more rational and predictive design strategies. Furthermore, scaling up production in accordance with Good Manufacturing Practice (GMP) standards, rigorously controlling key quality attributes such as drug loading, molecular weight, and purity, and establishing a clear regulatory pathway for “active carrier” systems constitute essential steps toward translating these promising natural PDCs into clinical applications. Table 3 provides a summary of the current opportunities for natural polymers in PDCs formation, together with the key challenges that research is still facing.

In conclusion, bioactive polymers constitute an exceptionally promising class of carriers for PDCs. Their inherent biocompatibility, biodegradability, and biological functions allow them to act synergistically with the therapeutic agents they carry, rather than passive scaffolds. When combined with smart conjugation chemistries (e.g., cleavable linkers) and precise polymer architectures, they can deliver controlled release, improved pharmacokinetics, and enhanced efficacy. With continued interdisciplinary efforts across polymer chemistry, pharmaceutical sciences, biomedicine, computational modeling, and process engineering, current limitations can be overcome, and natural PDCs are poised to become a cornerstone of next-generation precision therapies.

## Figures and Tables

**Table 1:** Summary of important factors in the choice of a polymer for PDC formulation and how they affect the final system.

	Factor	What It Influences	How It Affects the PDC	References
Polymer properties	Solubility	Drug formulation; Bioavailability; Systemic delivery.	Hydrophilic polymers enhance the aqueous solubility of poorly soluble drugs, improving dissolution, stability, and systemic delivery of hydrophobic therapeutics.	[25,30]
	Molecular Weight	Tumor accumulation; Circulation time; Renal clearance; Drug loading.	High-molecular-weight polymers often show prolonged plasma circulation and increased tumor accumulation, whereas lower molecular weight polymers may improve drug conjugation efficiency and cellular activity.	[31,32,33]
	Charge	Cellular uptake; Intracellular localization; Cytotoxicity.	Polymer charge affects interactions with cell membranes and biological barriers, influencing cellular internalization, cytotoxicity, and therapeutic performance.	[34,35]
	Polymer Architecture	Drug loading; Release behavior; Biodistribution; Circulation.	Structural features such as linear, branched, star-shaped, or block copolymer architectures influence drug conjugation efficiency, release profiles, and in vivo behavior of the conjugate.	[36,37,38,39]
	Functional Groups	Conjugation chemistry; Drug loading; Release kinetics.	Reactive groups on the polymer backbone (e.g., amines, hydroxyls, and thiols) determine which drugs can be attached, the efficiency of coupling reactions, and the resulting drug-release mechanisms.	[40,41]
PDC properties	Biocompatibility and Biodegradability	Safety profile; Toxicity; Immune response.	Biocompatible polymers minimize cytotoxicity, immunogenicity, and adverse tissue responses, while biodegradable polymers break down into non-toxic products that can be eliminated after drug release. This improves overall safety and reduces systemic toxicity.	[42,43,44]
	Pharmacokinetics	Circulation time; Biodistribution; Cellular uptake.	Polymer conjugation can extend circulation time, modify biodistribution, reduce off-target exposure, and enhance drug accumulation at target sites, improving therapeutic efficacy.	[45,46]

**Table 2:** Summary of different stimuli used in PDCs to achieve enhanced stability, increased efficacy, targeted drug release, or refined pharmacokinetics.

Stimulus	Polymer	Drug	Advantages	Reference
pH	HPMA copolymer	DOX	Increased stability and half-life; improved intracellular uptake; enhanced mitochondrial targeting when combined with R8–MTS peptide; stronger antimetastatic effect.	[130]
	Polyglutamic acid	DOX + AGM	Targeted drug release at acidic pH; enhanced antitumor efficacy; reduced cardiotoxicity; improved in vivo antitumor activity.	[131]
Redox	Dextran–PBA	Naproxen	Inflammation-specific release; reduction in IL-6/TNF-α; synergistic anti-inflammatory activity.	[132]
	Poly(disulfide) (PSS)	DOX	Selective intracellular activation; strong GSH depletion; enhanced tumor inhibition	[133]
Temperature	PropOzi–C2MestOx	Cefazolin	LCST-dependent phase transition; temperature-activated drug release; enhanced antibacterial activity.	[134]
	poly(NIPAM-co-AAm)	DOX	Dual temperature/pH responsiveness; selective and targeted drug release; cytotoxicity comparable to free DOX.	[135]

**Table 3:** Summary of the current opportunities and key challenges for natural polymers in PDC formation from the point of view of their natural origin, together with biological and immunological considerations. Additionally, opportunities and challenges in manufacturing and clinical translation are presented.

Category	Current Opportunities	Key Challenges
Material origin	Natural polymers offer advantages due to their natural availability, low cost, and easy manufacturing.	Variability from biological sources and the presence of impurities can affect solubility, degradation behavior, formulation stability, and drug-release profiles.
Biological evaluation	Advances in computational modeling and machine learning are enabling rational polymer design by correlating polymer structure, conjugation chemistry, and biological performance.	More systematic studies are required to understand biodistribution, immunogenicity, degradation pathways, and long-term safety.
Immunological considerations	Some synthetic polymers (e.g., PEG) provide established benefits due to their stealth properties and pharmacokinetic advantages.	Repeated administration may lead to anti-polymer antibodies, accelerated blood clearance, hypersensitivity, and allergic reactions, highlighting potential immunogenicity risks.
Manufacturing and standardization	Development of chemically defined analogues of natural polymers and controlled polymerization can improve uniformity and reduce batch-to-batch variability.	Achieving scalable production and strict structural control remains technically challenging. Newly developed synthetic polymers often lack comprehensive long-term biological and clinical safety data.
Translation and regulatory considerations	Progress in GMP-compliant manufacturing, quality control (drug loading, molecular weight, purity), and regulatory frameworks can support clinical translation.	Establishing standardized regulatory pathways for active carrier systems remains a major hurdle.

## Data Availability

No new data were created or analyzed in this study. Data sharing is not applicable to this article.
